# Editorial: Sensing of and acclimations to environmental pH in plants

**DOI:** 10.3389/fpls.2024.1447857

**Published:** 2024-07-11

**Authors:** Isabel Cristina Vélez-Bermúdez, Li Liu, Christoph-Martin Geilfus

**Affiliations:** ^1^ Institute of Plant and Microbial Biology, Academia Sinica, Taipei, Taiwan; ^2^ College of Life Sciences, Beijing Normal University, Beijing, China; ^3^ Department of Soil Science and Plant Nutrition, Hochschule Geisenheim University, Geiseneim, Germany

**Keywords:** alkaline soils, pH homeostasis, bicarbonate, iron acquisition, nitrate uptake, acclimation, plasma membrane, H+-ATPase

The discovery of pH sensors in the root apoplast of the reference plant *Arabidopsis thaliana* which can perceive and convey information on the external proton concentration demonstrated that plants can respond to changes in external pH by prioritising crucial processes such as growth or defence (Liu et al., 2022). During salinity for instance, the leaf apoplast pH (pH_apo_) transiently alkalizes, reducing stomatal aperture by increasing abscisic acid (ABA) levels. This pH change initiates stomatal closure, adjusts protein abundances, and stiffens the cell wall, thereby affecting growth. Proteomic analyses show that pH_apo_ influences growth-related proteins, linking chloride stress to reduced growth (Geilfus, 2017). It is quite unclear, however, by which mechanisms such cues are transduced to alter the expression of genes that ultimately aid plants in adapting to alterations in the pH of their environment. Soil pH is a master regulator that orchestrates a plethora of processes affecting plant performance and fitness, including but not limited to the activity of the microbiome, the composition of root exudates, the level of potentially toxic ions, and the availability of mineral nutrients (Tsai and Schmidt, 2021). The latter phenomenon has a pronounced impact on both the quality and yield of crop plants and on the composition of plant communities in natural ecosystems, leading to a distinction between species adapted to acid soil (so-called calcifuge species), and species that preferentially thrive in circumneutral or alkaline soils (calcicole species). Low pH favours the formation of cell-damaging aluminium species such as Al^3+^, limits the availability of phosphorous and nitrate, and increases the availability of manganese and iron, often to toxic levels. In alkaline soils, on the other hand, the availability of iron for plants dramatically declines, reducing its mobility by circa 1,000-fold for every one-digit increase in pH. Besides potential iron deficiency, alkaline soils are characterised by low available water capacity and limited supply of nitrate, phosphate, and manganese. The acquisition of iron in such soils is further restricted by the presence of bicarbonate (HCO_3_^−^), a typical feature of calcareous soils which strongly buffers soil pH and shifts apoplastic pH to higher than desired values. Bicarbonate adversely affects growth, photosynthesis and iron acquisition, likely by factors that are possibly associated with, but that are largely independent of pH *per se*.

In the present Research Topic, Bailey et al. took up the challenge to distinguish between effects exerted by bicarbonate, iron deficiency, pH, and nitrate starvation by comparing published transcriptomic data sets with an RNA-seq-derived transcriptome derived from roots of plants subjected to short-term exposure to elevated pH. Co-expression analysis of genes that are differentially expressed under the above conditions identified a suite of genes with putative functions in root growth under alkaline conditions, a cluster that appears to be conserved in the transcriptome of plants exposed to low pH (albeit with opposite direction of expression) or bicarbonate. The authors further identified alterations in anion/H^+^-coupled transport as a means to enrich protons inside the cell and to recalibrate cytosolic pH in response to exposure to high pH. The study allowed the conclusion that bicarbonate but not alkalinity as such triggers an iron deficiency response, suggesting separate effects of the two stressors.

A similar conclusion derived from the study by Zhao et al. *Cydonia oblonga* Mill. (quince A) is a chlorosis-sensitive rootstock used in pear cultivation. The authors found that, in contrast to the iron-efficient rootstock *Pyrus betulifolia* (PB), quince A developed iron deficiency symptoms when exposed to bicarbonate, an effect that was not observed when the plants were exposed to high pH without bicarbonate. Differences between alkalinity as such and bicarbonate were also observed for root growth, which was promoted by high pH but ceased when plants encountered the combinatory effects of high pH and bicarbonate.

The electrochemical gradient across membranes is an essential feature that drives transport processes across cellular boundaries. Ions crossing the plasma membrane alter this gradient, potentially affecting critical processes such as transport, growth, and defence. Changes in external pH towards more acidic or more alkaline conditions recruit specific nitrate transporters, the expression of which is typically induced by high or low proton concentrations, suggesting that the transport of protons associated with nitrate uptake plays a critical role in the homeostasis of cytosolic and apoplastic pH (Bailey et al.). In a study on Arabidopsis, Sena and Kunze show that the nitrate transporter NRT1.5, localised to the plasma membrane of root pericycle cells, is binding to one of the major proton pumps in the plasma membrane, the P-type ATPase AHA2. The authors further report that together with AHA2, NRT1.5 mediates proton-coupled root-to-shoot transport of both K^+^ and NO_3_^-^ ions by loading the nutrients into the xylem of root cells. The authors conclude that the interplay between the two transporters not only controls the distribution of K^+^ and NO_3_^-^ within the plants, but also is crucial for the plant’s H^+^ homeostasis.

Vélez-Bermúdez and Schmidt summarise what is currently known regarding the mechanisms and consequences of pH sensing in plants. Exposure to either acidic or alkaline pH appear to trigger distinct peptide-ligand interactions, which in turn induce pronounced changes in transcriptomic and proteomic profiles. Surprisingly, not only roots but also cells that have no direct exposure to the soil appear to be able to sense and respond to external pH changes in the media, suggesting intricate communication pathways among plant tissues and organs. In their Opinion paper, the authors picture environmental pH as a major determinant of phenotypic plasticity that modulate growth, defence, and development of the plant and suppose that the mechanisms involved in pH sensing and its coupling with the myriads of signalling cascades and their functional readouts is far from being completely understood.

This overview suggests that external pH is a largely underestimated factor in plant function and adaptation. Protons play a significant role in defining and prioritizing plant responses and developmental processes, a relationship that has just begun to be explored. Sensing and acclimating to environmental pH are crucial elements in the chain of events that govern plant phenotypical plasticity. External pH is vital for plant growth, regulating nutrient availability, microbial activity, and toxic ion levels.

## Author contributions

ICV-B: Writing – original draft. LL: Writing – review & editing. C-MG: Writing – review & editing.
